# Behandlung der posttraumatischen Belastungsstörung – die aktualisierte S3-Leitlinie

**DOI:** 10.1007/s00115-026-02003-z

**Published:** 2026-08-13

**Authors:** Ingo Schäfer, Thomas Ehring, Ulrich Frommberger, Britta Jacobsen, Christine Knaevelsrud, Astrid Lampe, Kirsten Lehmann, Cosima Leithner, Andreas Maercker, Paul Plener, Rita Rosner, Bianca Schreyer, Carsten Spitzer

**Affiliations:** 1 Klinik für Psychiatrie und Psychotherapie, Universitätsklinikum Hamburg-Eppendorf, Martinistr. 52, 20246 Hamburg, Deutschland; 2 Lehrstuhl für Klinische Psychologie und Psychotherapie, LMU München, München, Deutschland; 3 Privatpraxis, Sölden, Deutschland; 4 FB Erziehungswissenschaft und Psychologie, Arbeitsbereich Klinisch-Psychologische Intervention, Freie Universität Berlin, Berlin, Deutschland; 5 Ludwig-Boltzmann-Institut für Rehabilitationsforschung, Wien, Österreich; 6 Psychologisches Institut, Fachrichtung Psychopathologie und Klinische Intervention, Universität Zürich, Zürich, Schweiz; 7 Universitätsklinik für Kinder- und Jugendpsychiatrie, Medizinische Universität Wien, Wien, Österreich; 8 Klinische und Biologische Psychologie, Katholische Universität Eichstätt-Ingolstadt, Ingolstadt, Deutschland; 9 Klinik für Psychosomatische Medizin und Psychotherapie, Universitätsmedizin Rostock, Rostock, Deutschland; 10 Deutsches Zentrum für Psychische Gesundheit (DZPG), Standortverbund Berlin–Potsdam, Berlin–Potsdam, Deutschland; 11 Rehaklinik Montafon, Bereich Psychische Gesundheit, Schruns, Österreich

**Keywords:** Trauma, Psychotherapie, Internettherapie, Pharmakotherapie, Traumainformierte Behandlung, Trauma, Psychotherapy, Internet-based therapy, Pharmacotherapy, Trauma-informed treatment

## Abstract

Die S3-Leitlinie zur posttraumatischen Belastungsstörung (PTBS) wurde unter Federführung der Deutschsprachigen Gesellschaft für Psychotraumatologie (DeGPT) aktualisiert und erweitert. Aufgrund ihrer hohen Prävalenz sollen traumatische Erfahrungen bei jeder Befund- und Anamneseerhebung erfasst werden. Die traumafokussierte Psychotherapie bleibt Therapie der ersten Wahl und soll auch bei der komplexen PTBS (kPTBS) mit komorbiden Störungen angeboten werden sowie bei älteren Menschen, Menschen mit Fluchthintergrund und Menschen mit leichten bis mittelgradigen Störungen der Intelligenzentwicklung. Inzwischen liegt zu einem noch breiteren Spektrum von Methoden überzeugende Evidenz vor, insbesondere aus dem Bereich der kognitiven Verhaltenstherapie (KVT). Traumafokussierte Interventionen können als Onlinebehandlung und in höherer Frequenz (mehrfach pro Woche) angeboten werden. Auch bei Kindern und Jugendlichen ist die traumafokussierte KVT die Therapie der Wahl. Medikamente sind eine Behandlung zweiter Wahl und werden bei Kindern und Jugendlichen nicht empfohlen. Benzodiazepine und Z‑Subtanzen sollen generell nicht eingesetzt werden. Unter den adjuvanten Verfahren existiert die beste Evidenz zu Sport- und Bewegungstherapie. Ein wichtiges allgemeines Behandlungsprinzip betrifft die Etablierung traumainformierter Grundprinzipien („trauma-informed care“) als standardisiertem Ansatz in Einrichtungen des Gesundheitswesens.

## Abstract (en)

The S3 guidelines on posttraumatic stress disorder (PTSD) have been updated and expanded under the leadership of the German-speaking Society for Psychotraumatology (DeGPT). Given the high prevalence of trauma exposure, traumatic experiences should be assessed during all clinical evaluations and history taking. Trauma-focused psychotherapy remains the first-line treatment and should also be offered in cases of complex PTSD (cPTSD) with comorbid disorders, in older people, refugees and individuals with mild to moderate intellectual disabilities. Strong evidence is now available for a broader range of interventions, particularly cognitive behavioral therapy (CBT). Trauma-focused interventions can be delivered online and at higher intensity (several sessions per week). For children and adolescents, trauma-focused CBT also remains the treatment of choice. Pharmacotherapy is the second-line treatment and is not recommended for children or adolescents. Benzodiazepines and Z‑drugs should generally be avoided. Among adjuvant forms of treatment the strongest evidence is available for exercise and physical activity. An important treatment principle involves the establishment of trauma-informed basic principles (trauma-informed care) as a standard approach in institutions of the healthcare system.

## Hintergrund

Die posttraumatische Belastungsstörung (PTBS) gehört zu den häufigen psychischen Erkrankungen mit erheblicher individueller und gesellschaftlicher Relevanz. Ein Großteil der Bevölkerung erlebt im Laufe des Lebens potenziell traumatische Ereignisse. In deren Folge wird für Deutschland eine Einmonatsprävalenz der Störung von 1,5 % berichtet. In bestimmten Risikogruppen, etwa bei Personen mit Flucht- und Migrationserfahrungen, tritt die PTBS wesentlich häufiger auf.

Die Störung ist häufig mit deutlichen funktionellen Einschränkungen und einer reduzierten Lebensqualität, aber auch mit somatischen Erkrankungen, etwa im kardiovaskulären, muskuloskelettalen und immunologischen Bereich, assoziiert [6]. Zudem besteht eine hohe psychiatrische Komorbidität [4, 12]. Insgesamt ist die PTBS mit hohen direkten und indirekten Krankheitskosten verbunden, unter anderem durch Arbeitsunfähigkeit, Frühberentung und reduzierte Erwerbsfähigkeit [15].

Aufgrund neuer Erkenntnisse zur Diagnostik und Behandlung der PTBS ergab sich ein Aktualisierungsbedarf der bestehenden S3-Leitlinie. Sie wurde grundlegend überarbeitet, die Evidenzlage systematisch neu bewertet und neue Empfehlungen für unterschiedliche Versorgungsbereiche und Patient:innengruppen formuliert.

## Revisionsprozess

Die Revision der Leitlinie wurde unter Federführung der Deutschsprachigen Gesellschaft für Psychotraumatologie (DeGPT) von 45 Fachgesellschaften und Institutionen sowie Betroffenenvertretungen durchgeführt [13]. Ein zentraler methodischer Punkt war die konsequente Umstellung der Evidenzbewertung auf das GRADE-System [5]. Darüber hinaus wurde die Leitlinie inhaltlich erweitert. Erstmals wurden Gruppen mit besonderen Bedarfen, darunter ältere Menschen, Personen mit intellektuellen Beeinträchtigungen sowie Menschen mit Fluchthintergrund, systematisch berücksichtigt. Zudem wurden Empfehlungen zu allgemeinen Behandlungsprinzipien formuliert. Die Konsensfindung erfolgte in Onlinekonferenzen unter Moderation durch die Arbeitsgemeinschaft der Wissenschaftlichen Medizinischen Fachgesellschaften (AWMF; [13]).

## Empfehlungen der überarbeiteten Leitlinie

Die überarbeitete Leitlinie umfasst 11 Kapitel mit 70 Empfehlungen. Die Empfehlungen zur Diagnostik, Psychotherapie, Pharmakotherapie, komplexen PTBS und Komorbidität werden im Folgenden ausführlicher dargestellt, die anderen Kapitel inhaltlich zusammengefasst. Die vollständigen Empfehlungen können der Druckversion der Leitlinie entnommen werden [13].

### Empfehlungen zur Diagnostik

Die Empfehlungen betonen die zentrale Bedeutung einer aktiven Erfassung traumatischer Erfahrungen (Tab. 1). Da Betroffene traumatische Erlebnisse und damit verbundene Symptome häufig nicht spontan berichten [3], soll eine Traumaanamnese regelhaft Bestandteil der psychodiagnostischen Befund- und Anamneseerhebung sein.

**Tab. 1:** Empfehlungen zur Diagnostik (Auswahl)

Nr	Empfehlung	Empfehlungskategorie (Konsens %)
1.1	Bei jeder psychodiagnostischen Befund- und Anamneseerhebung im Erwachsenenalter soll erfasst werden, ob ein potenziell traumatisches Ereignis in der Lebensgeschichte erlebt wurde	EK (95,8)
1.2	Wenn mindestens ein potenziell traumatisches Ereignis berichtet wird oder unklar ist, ob potenziell traumatische Ereignisse in der Lebensgeschichte erlebt wurden, soll eine differenzierte Traumaanamnese erfolgen	EK (100)
1.3	Wenn keine differenzierte Traumaanamnese möglich ist, sollen PTBS-Screeninginstrumente eingesetzt werden	EK (96,0)
1.4	Beim Vorliegen mindestens eines potenziell traumatischen Ereignisses soll zur Unterstützung der (k)PTBS-Diagnostik ein strukturiertes Interview angewandt werden oder die Erfassung der posttraumatischen Symptomatik mittels eines validierten Selbstberichtsinstrumentes erfolgen. Wenn möglich, ist ein strukturiertes Interview zu bevorzugen	EK (87,5)

*EK* Expert:innenkonsens, *(k)PTBS* (komplexe) posttraumatische Belastungsstörung

**Tab. 2:** Empfehlungen zur Psychotherapie bei posttraumatischen Belastungsstörungen

Nr	Empfehlung	Empfehlungskategorie bzw. -grad (Konsens %)
2.1	Patient:innen mit einer posttraumatischen Belastungsstörung soll eine traumafokussierte Psychotherapie* im Einzelsetting angeboten werden, bei der der Schwerpunkt auf der Auseinandersetzung mit den traumatischen Ereignissen und den dazugehörigen Kognitionen, Emotionen und Erinnerungen liegt	EE/A (92,0)
2.2	Für eine generell notwendige vorausgehende Stabilisierungsphase vor einer traumafokussierten Psychotherapie liegt derzeit für erwachsene Patient:innen mit einer PTBS keine Evidenz vor	Statement (90,0)
2.3	Bei Präferenz kann Psychotherapie (einschließlich traumafokussierter Psychotherapie, s. Empfehlung 2.1) für PTBS-Patient:innen auch unter Nutzung zertifizierter Videodienstanbieter erwogen werden. Dabei sind Rahmenbedingungen, Indikation sowie Risiken und Nebenwirkungen der videogestützten Therapie zu berücksichtigen	EK (96,0)
2.4	Traumafokussierte Psychotherapie für PTBS kann Patient:innen auch in höherer Frequenz (d. h. mehrfach pro Woche) angeboten werden	EK (100)

*EE* evidenzbasierte Empfehlung; *EK* Expert:innenkonsens; *A* Soll-Empfehlung, *PTBS* Posttraumatische Belastungsstörung

*Insbesondere folgende Interventionen werden aufgrund der robusten Evidenz empfohlen (alphabetische Aufzählung): Eye Movement Desensitization and Reprocessing (EMDR), kognitive Therapie (KT), kognitive Verarbeitungstherapie (Cognitive Processing Therapy, CPT), narrative Expositionstherapie (NET), prolongierte Exposition (PE)

Weiter empfiehlt die Leitlinie den Einsatz validierter, strukturierter Instrumente (z. B. [1, 17]). Interviews, die in den Empfehlungen genannt werden, sind das *International Trauma Inventory* (ITI) und die *Clinician-Administered PTSD Scale for DSM‑5* (CAPS-5). Als Selbstbeurteilungsinstrumente werden die *PTSD Checklist for DSM‑5* (PCL-5), die *PTSD Diagnostic Scale for DSM‑5* (PDS-5) und das *International Trauma Questionnaire* (ITQ) genannt.

### Empfehlungen zur Psychotherapie

Es existiert eine starke Evidenz dazu, dass traumafokussierte Methoden nichttraumafokussierten Ansätzen überlegen sind (z. B. [7, 18]). Dies betrifft verschiedene Varianten der traumafokussierten kognitiven Verhaltenstherapie (tf-KVT) sowie Eye Movement Desensitization and Reprocessing (EMDR). Für ein generelles phasenbasiertes Vorgehen findet sich für die klassische PTBS keine ausreichende Evidenz.

Videogestützte Psychotherapie zeigt in Metaanalysen vergleichbare Effekte [14]. Höherfrequente Behandlungen sind mindestens so wirksam sind wie Standardtherapien, bei schnellerer Besserung und weniger Therapieabbrüchen ([8]; Tab. 2).

### Empfehlungen zur Pharmakotherapie

Pharmakotherapie kann wirksam sein, ihre Effekte sind jedoch geringer als die einer traumafokussierten Therapie, sodass sie eine Behandlung der zweiten Wahl darstellt. Die beste Evidenz existiert für Sertralin, Paroxetin, Fluoxetin und Venlafaxin (Fluoxetin und Venlafaxin: off-label), mit kleinen bis moderaten Effekten (z. B. [9, 16]). Für Albträume können α1-Adrenozeptor-Antagonisten wirksam sein (off-label; [11]).

Benzodiazepine und sog. „Z-Substanzen“ (Zopiclon, Zolpidem) weisen ein ungünstiges Nutzen-Risiko-Profil auf und sollen nicht eingesetzt werden. Insgesamt werden Medikamente als ergänzende Option im Rahmen eines Gesamtkonzepts eingeordnet (Tab. 3).

**Tab. 3:** Empfehlungen zur pharmakologischen Behandlung der posttraumatischen Belastungsstörung

Nr	Empfehlung	Empfehlungskategorie bzw. -grad (Konsens %)
3.1	Bei der Behandlung der PTBS soll primär eine traumafokussierte Psychotherapie angeboten werden. Eine Pharmakotherapie ist die Behandlung der zweiten Wahl, beispielsweise bei Patient:innenpräferenz, unzureichendem Ansprechen oder fehlender Verfügbarkeit einer traumafokussierten Psychotherapie	EE/A (92,6)
3.2	Eine psychopharmakologische Behandlung der PTBS soll mit Sertralin, Paroxetin, Fluoxetin* oder Venlafaxin* erfolgen	EE/A (88,0)
3.3	Eine psychopharmakologische Behandlung von Albträumen im Rahmen einer PTBS kann mit Prazosin* erwogen werden	EE/0 (82,6)
3.4	Im Rahmen einer traumafokussierten Psychotherapie sollte eine Pharmakotherapie nur zur Behandlung von Schlafstörungen oder komorbiden Störungen angeboten werden	EK (86,4)
3.5	Benzodiazepine und Z‑Substanzen sollen nicht zur Behandlung der PTBS eingesetzt werden	EK (95,7)

*EE* evidenzbasierte Empfehlung; *EK* Expert:innenkonsens; *A* Soll-Empfehlung; *0* Kann-Empfehlung, *PTBS* Posttraumatische Belastungsstörung

*Off-label-Verordnung

**Tab. 4:** Empfehlungen zur Behandlung der komplexen posttraumatischen Belastungsstörung

Nr	Empfehlung	Empfehlungskategorie/-grad (Konsens %)
4.1	Für eine kPTBS nach ICD-11 sollte die psychotherapeutische Behandlung mit einer Kombination traumafokussierter Techniken erfolgen, bei denen Schwerpunkte auf der Verarbeitung der Erinnerung an die traumatischen Erlebnisse und/oder ihrer Bedeutung liegen sowie auf Techniken zur Emotionsregulation und zur Verbesserung des Selbstwertgefühls sowie von Beziehungsstörungen im Sinne der Bearbeitung dysfunktionaler zwischenmenschlicher Muster	EE/B (90,9)
4.2	Ein mehrere Phasen umfassendes Vorgehen sollte einem ausschließlich isoliert traumafokussierten Vorgehen vorgezogen werden	EK (91,3)
4.3	Psychotherapie für kPTBS kann auch in höherer Frequenz (d. h. mehrfach in der Woche) angeboten werden	EK (96,0)

*EE* evidenzbasierte Empfehlung; *EK* Expert:innenkonsens; *B* Sollte-Empfehlung, *ICD-10* International Statistical Classification of Diseases and Related Health Problems 10, *PTBS* Posttraumatische Belastungsstörung

### Empfehlungen zur Behandlung der komplexen PTBS

Die Evidenz zur kPTBS ist noch begrenzt, aber es zeigt sich, dass traumafokussierte Methoden auch bei dieser Gruppe von Patient:innen wirksam sind (z. B. [2, 10]).

Zudem gibt es Hinweise darauf, dass kombinierte Behandlungsansätze, die traumafokussierte Interventionen mit Techniken zur Emotionsregulation und zur Bearbeitung interpersoneller Probleme verbinden, bei der kPTBS oft das sinnvollere Vorgehen darstellen (Tab. 4).

### Empfehlungen zu Komorbiditäten bei PTBS

Psychische Komorbiditäten sind bei der PTBS die Regel. Sie umfassen insbesondere affektive Störungen, Angststörungen, Substanzgebrauchsstörungen sowie Persönlichkeitsstörungen. Traumafokussierte Psychotherapie ist auch bei Patient:innen mit komorbiden Störungsbildern wirksam, sicher und anderen Behandlungsansätzen überlegen [4, 12].

Komorbide Störungen stellen grundsätzlich keine Kontraindikation für traumafokussierte Interventionen dar, können jedoch im Einzelfall, etwa bei ausgeprägten Störungen der Verhaltenskontrolle, eine initiale Stabilisierung erforderlich machen (Tab. 5).

**Tab. 5:** Empfehlungen zur Behandlung bei Komorbidität

Nr	Empfehlung	Empfehlungskategorie bzw. -grad (Konsens %)
6.1	Patient:innen mit einer PTBS und komorbiden psychischen Störungen soll eine traumafokussierte Psychotherapie im Einzelsetting angeboten werden, bei der der Schwerpunkt auf der Auseinandersetzung mit den traumatischen Ereignissen und den dazugehörigen Kognitionen, Emotionen und Erinnerungen liegt	EE/A (100)
6.2	Bei der Behandlung der (k)PTBS sollte die klinische Komorbidität in einem Gesamtbehandlungsplan mit „partizipativer Entscheidungsfindung“ berücksichtigt werden	EK (82,6)
6.3	Auch Patient:innen mit (k)PTBS und potenziell gefährdenden Symptomen (z. B. Suizidalität, psychotische Symptome, Selbstverletzung, Fremdaggression, Substanzkonsum) soll, sofern diese nicht mit schwerwiegenden Störungen der Verhaltenskontrolle einhergehen, eine traumafokussierte Psychotherapie angeboten werden	EK (100)
6.4	Wenn bei Patient:innen mit (k)PTBS aktuell schwerwiegende Störungen der Verhaltenskontrolle vorliegen, sollen diese zunächst behandelt und eine traumafokussierte Psychotherapie im Verlauf angeboten werden	EK (100)

*EE* evidenzbasierte Empfehlung; *EK* Expert:innenkonsens; *A* Soll-Empfehlung, *(k)PTBS* (komplexe) posttraumatische Belastungsstörung

### Empfehlungen zur adjuvanten Behandlung

Die Leitlinie empfiehlt insbesondere den Einsatz von Sport- und Bewegungstherapie, für die eine (wenn auch niedrige) Evidenzlage zu positiven Effekten auf PTBS- und depressive Symptome besteht. Weitere Verfahren wie Physio‑, Sozio- und Ergotherapie sowie künstlerische Therapien werden auf Basis klinischer Erfahrung empfohlen, insbesondere im Rahmen multimodaler, traumaspezifischer Behandlungskonzepte. Gleichzeitig wird betont, dass weitere Verfahren (z. B. neurobiologische oder alternativmedizinische Ansätze) zunehmend untersucht werden, aber noch nicht ausreichend bewertet werden können.

### Empfehlungen zu älteren Menschen

Die Evidenzlage zur Behandlung der PTBS im höheren Lebensalter ist begrenzt. Es finden sich jedoch keine Hinweise darauf, dass traumafokussierte Methoden bei älteren Patient:innen weniger wirksam sind als bei jüngeren. Entsprechend wird empfohlen, auch im höheren Lebensalter primär eine traumafokussierte Psychotherapie anzubieten. Pharmakotherapie stellt, analog zu jüngeren Erwachsenen, die Therapie der zweiten Wahl dar, wobei altersbedingte Risiken (z. B. Nebenwirkungen) besonders zu berücksichtigen sind. Zusätzlich werden traumainformierter Pflegekonzepte hervorgehoben.

### Empfehlungen zu Menschen mit Fluchthintergrund

Auch für geflüchtete Menschen stellt die traumafokussierte Psychotherapie die Behandlung der ersten Wahl dar. Die Notwendigkeit einer kultursensiblen Diagnostik unter Verwendung validierter und übersetzter Instrumente sowie die Berücksichtigung psychosozialer Kontextfaktoren wird betont. Zudem wird der Einsatz qualifizierter Sprachmittler:innen als essenziell für eine adäquate psychotherapeutische Versorgung hervorgehoben.

### Empfehlungen zu Menschen mit Störungen der Intelligenzentwicklung

Zentrale Herausforderungen bestehen in der Diagnostik und der insgesamt sehr begrenzten Evidenzlage. Die Leitlinie empfiehlt diagnostische Verfahren an den kognitiven und emotionalen Entwicklungsstand anzupassen und fremdanamnestische Informationen einzubeziehen. Bei leichter bis mittelgradiger Ausprägung werden traumafokussierte Methoden empfohlen, wobei sie strukturell und inhaltlich adaptiert werden müssen. Pharmakotherapie soll primär bei Therapieresistenz eingesetzt werden.

### Empfehlungen zu Kindern und Jugendlichen

#### Diagnostik

Zentral ist erneut, dass eine Traumaanamnese Bestandteil jeder diagnostischen Abklärung sein soll. PTBS und kPTBS sollen unter Berücksichtigung entwicklungsabhängiger Symptome diagnostiziert werden, ergänzt durch valide, altersadaptierte Instrumente.

#### Psychotherapie

Die traumafokussierte Psychotherapie ist die Behandlung der ersten Wahl, auch bei komplexer PTBS. Evidenz liegt vor allem zur traumafokussierten kognitiven Verhaltenstherapie (TF-KVT) vor, positive Befunde auch zu EMDR und der narrativen Expositionstherapie. Bei unzureichendem Ansprechen können ergänzende Interventionen erwogen werden.

#### Pharmakotherapie

Zu Psychopharmaka bei Kindern und Jugendlichen mit PTBS liegt keine ausreichende Evidenz vor, eine entsprechende Behandlung wird nicht empfohlen. Eine pharmakologische Therapie kann jedoch bei komorbiden Störungen indiziert sein.

#### Bezugspersonen und Setting

Der Einbezug von Bezugspersonen wird als wichtiger Bestandteil der Behandlung hervorgehoben. Zur Gruppentherapie liegen positive Befunde vor und sie erscheint insbesondere sinnvoll, wenn keine Einzeltherapie verfügbar ist.

### Empfehlungen zu allgemeinen Behandlungsprinzipien

Das Kapitel bündelt Grundsätze zur Versorgung von Menschen mit (k)PTBS und behandelt partizipative Entscheidungsfindung, Aufklärung, traumainformierte Versorgung sowie sektorenübergreifende Vernetzung. Hausärzt:innen kommt eine zentrale Rolle zu, da sie häufig erste Ansprechpersonen sind und den Behandlungsverlauf begleiten. Bei Hinweisen auf Partnerschaftsgewalt soll das spezialisierte Fachberatungssystem einbezogen werden. In der Peripartalphase ist auf Gewalterfahrungen und (k)PTBS-Symptome zu achten. Bei kraniomandibulärer Dysfunktion und Bruxismus soll eine zahnärztliche Mitbehandlung erfolgen. Zudem werden rehabilitative Maßnahmen bei fortbestehenden Teilhabeeinschränkungen, transparente Aufklärung bei Gerichtsverfahren sowie die Etablierung von „trauma-informed care“ als standardisierter Ansatz im Gesundheitswesen adressiert.

## Fazit für die Praxis

- Traumatische Erfahrungen sollen bei jeder Anamnese erfasst werden.
- Traumafokussierte Psychotherapie ist die Behandlung der ersten Wahl, auch bei komplexer PTBS und komorbiden Störungen.
- Bei komplexer PTBS sollte ein phasenbasiertes Vorgehen bevorzugt werden.
- Traumafokussierte Therapie kann auch online und in höherer Frequenz eingesetzt werden.
- Pharmakotherapie ist eine Behandlung der zweiten Wahl.
- Benzodiazepine und Z‑Substanzen sollen nicht eingesetzt werden.
- Sport und Bewegungstherapie ist die adjuvante Option mit der besten Evidenz.
- Auch älteren Menschen, Menschen mit Fluchthintergrund und Menschen mit Störung der Intelligenzentwicklung sollen traumafokussierte Behandlungen angeboten werden.
- „Trauma-informed care“ soll allgemein im Gesundheitswesen als zentraler Ansatz etabliert werden.

## Data Availability

Alle dieser Arbeit zugrunde liegenden Daten sind  unter https://register.awmf.org/de/leitlinien/detail/155-001 zugänglich.
